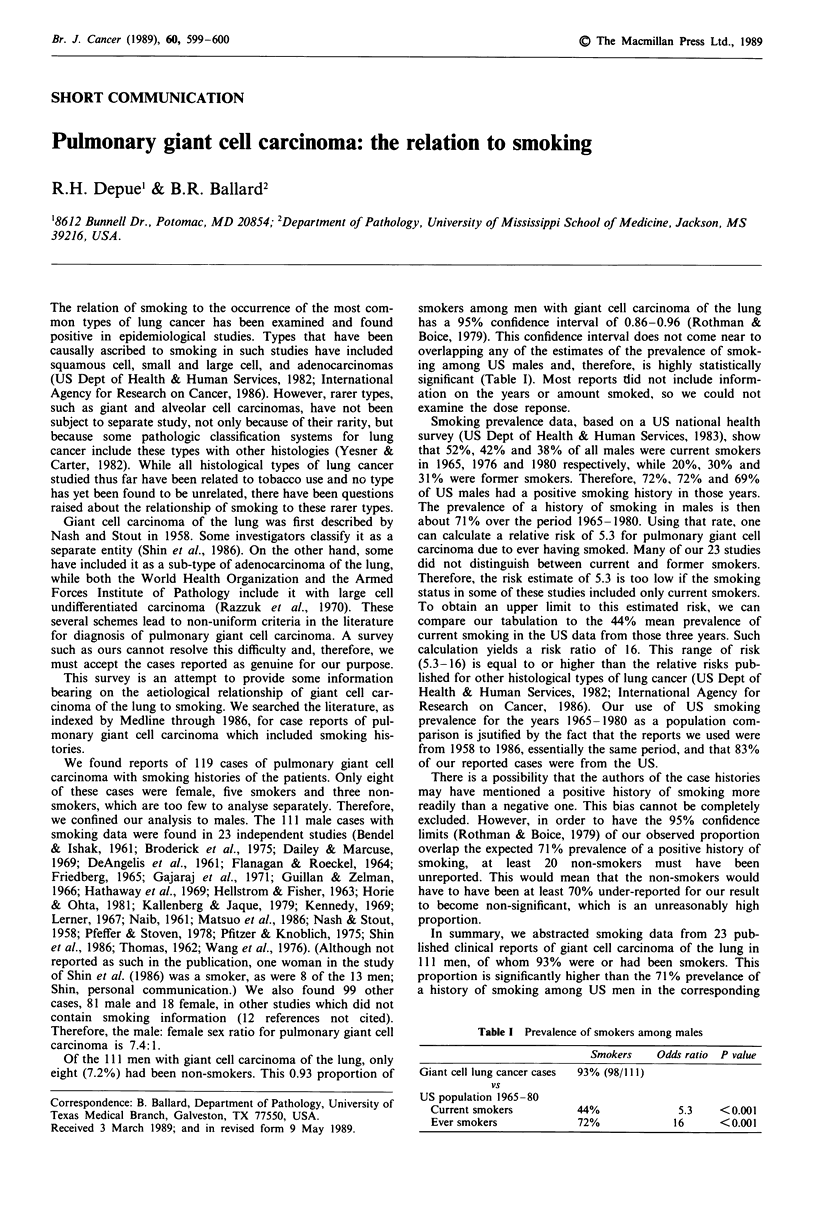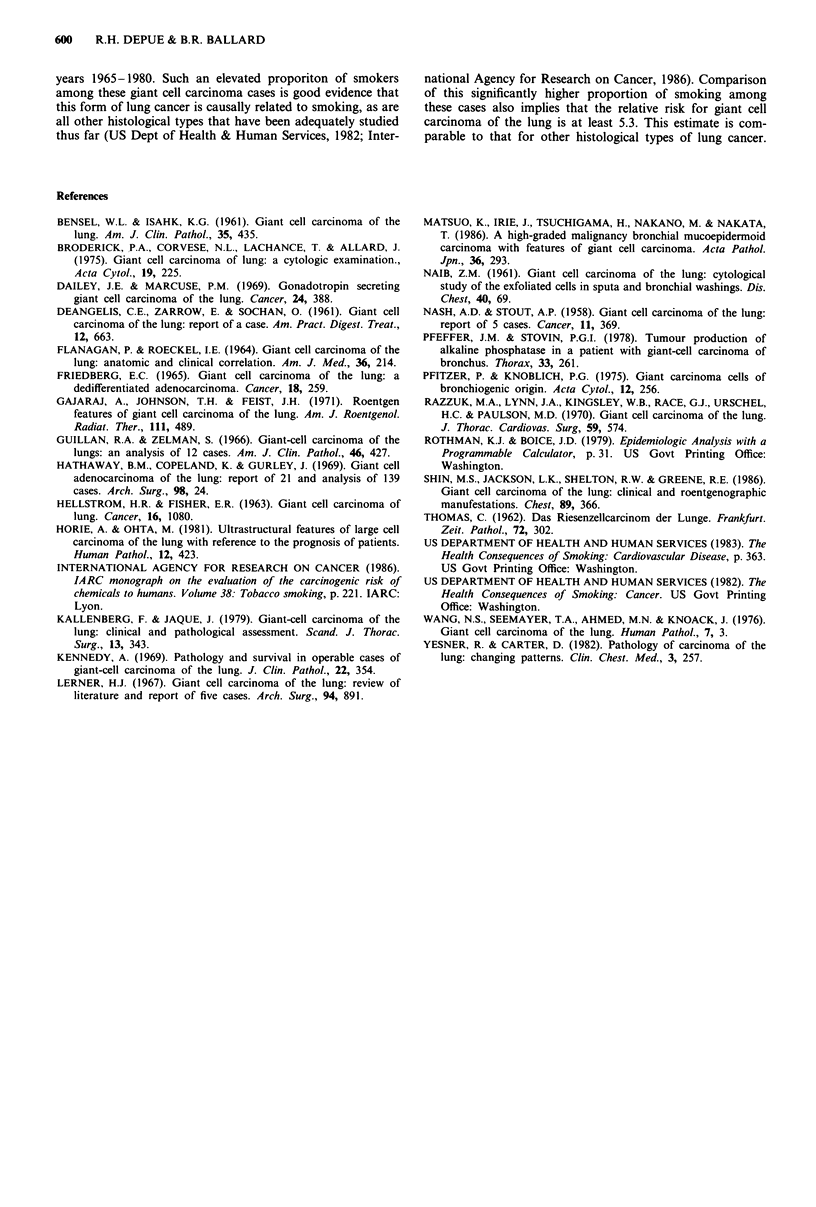# Pulmonary giant cell carcinoma: the relation to smoking.

**DOI:** 10.1038/bjc.1989.321

**Published:** 1989-10

**Authors:** R. H. Depue, B. R. Ballard

**Affiliations:** Department of Pathology, University of Mississippi School of Medicine, Jackson 39216.


Br. J. Cancer (1989), 60, 599-600 © The Macmillan Press Ltd., 1989

SHORT COMMUNICATION

### Pulmonary giant cell carcinoma: the relation to smoking

R.H. Depue[1] & B.R. Ballard[2]

[1]8612 Bunnell Dr., Potomac, MD 20854; [2]Department of Pathology, University of Mississippi School of Medicine, Jackson, MS 39216, USA.

The relation of smoking to the occurrence of the most common types of lung cancer has been examined and found positive in epidemiological studies. Types that have been causally ascribed to smoking in such studies have included squamous cell, small and large cell, and adenocarcinomas (US Dept of Health & Human Services, 1982; International Agency for Research on Cancer, 1986). However, rarer types, such as giant and alveolar cell carcinomas, have not been subject to separate study, not only because of their rarity, but because some pathologic classification systems for lung cancer include these types with other histologies (Yesner & Carter, 1982). While all histological types of lung cancer studied thus far have been related to tobacco use and no type has yet been found to be unrelated, there have been questions raised about the relationship of smoking to these rarer types.

Giant cell carcinoma of the lung was first described by Nash and Stout in 1958. Some investigators classify it as a separate entity (Shin *et al*., 1986). On the other hand, some have included it as a sub-type of adenocarcinoma of the lung, while both the World Health Organization and the Armed Forces Institute of Pathology include it with large cell undifferentiated carcinoma (Razzuk *et al*., 1970). These several schemes lead to non-uniform criteria in the literature for diagnosis of pulmonary giant cell carcinoma. A survey such as ours cannot resolve this difficulty and, therefore, we must accept the cases reported as genuine for our purpose.

This survey is an attempt to provide some information bearing on the aetiological relationship of giant cell carcinoma of the lung to smoking. We searched the literature, as indexed by Medline through 1986, for case reports of pulmonary giant cell carcinoma which included smoking histories.

We found reports of 119 cases of pulmonary giant cell carcinoma with smoking histories of the patients. Only eight of these cases were female, five smokers and three non-smokers, which are too few to analyse separately. Therefore, we confined our analysis to males. The 111 male cases with smoking data were found in 23 independent studies (Bendel & Ishak, 1961; Broderick *et al*., 1975; Dailey & Marcuse, 1969; DeAngelis *et al*., 1961; Flanagan & Roeckel, 1964; Friedberg, 1965; Gajaraj *et al*., 1971; Guillan & Zelman, 1966; Hathaway *et al*., 1969; Hellstrom & Fisher, 1963; Horie & Ohta, 1981; Kallenberg & Jaque, 1979; Kennedy, 1969; Lerner, 1967; Naib, 1961; Matsuo *et al*., 1986; Nash & Stout, 1958; Pfeffer & Stoven, 1978; Pfitzer & Knoblich, 1975; Shin *et al*., 1986; Thomas, 1962; Wang *et al*., 1976). (Although not reported as such in the publication, one woman in the study of Shin *et al*. (1986) was a smoker, as were 8 of the 13 men; Shin, personal communication.) We also found 99 other cases, 81 male and 18 female, in other studies which did not contain smoking information (12 references not cited). Therefore, the male: female sex ratio for pulmonary giant cell carcinoma is 7.4:1.

Of the 111 men with giant cell carcinoma of the lung, only eight (7.2%) had been non-smokers. This 0.93 proportion of smokers among men with giant cell carcinoma of the lung has a 95% confidence interval of 0.86–0.96 (Rothman & Boice, 1979). This confidence interval does not come near to overlapping any of the estimates of the prevalence of smoking among US males and, therefore, is highly statistically significant (Table I). Most reports did not include information on the years or amount smoked, so we could not examine the dose reponse.

Smoking prevalence data, based on a US national health survey (US Dept of Health & Human Services, 1983), show that 52%, 42% and 38% of all males were current smokers in 1965, 1976 and 1980 respectively, while 20%, 30% and 31% were former smokers. Therefore, 72%, 72% and 69% of US males had a positive smoking history in those years. The prevalence of a history of smoking in males is then about 71% over the period 1965–1980. Using that rate, one can calculate a relative risk of 5.3 for pulmonary giant cell carcinoma due to ever having smoked. Many of our 23 studies did not distinguish between current and former smokers. Therefore, the risk estimate of 5.3 is too low if the smoking status in some of these studies included only current smokers. To obtain an upper limit to this estimated risk, we can compare our tabulation to the 44% mean prevalence of current smoking in the US data from those three years. Such calculation yields a risk ratio of 16. This range of risk (5.3–16) is equal to or higher than the relative risks published for other histological types of lung cancer (US Dept of Health & Human Services, 1982; International Agency for Research on Cancer, 1986). Our use of US smoking prevalence for the years 1965–1980 as a population comparison is jsutified by the fact that the reports we used were from 1958 to 1986, essentially the same period, and that 83% of our reported cases were from the US.

There is a possibility that the authors of the case histories may have mentioned a positive history of smoking more readily than a negative one. This bias cannot be completely excluded. However, in order to have the 95% confidence limits (Rothman & Boice, 1979) of our observed proportion overlap the expected 71% prevalence of a positive history of smoking, at least 20 non-smokers must have been unreported. This would mean that the non-smokers would have to have been at least 70% under-reported for our result to become non-significant, which is an unreasonably high proportion.

In summary, we abstracted smoking data from 23 published clinical reports of giant cell carcinoma of the lung in 111 men, of whom 93% were or had been smokers. This proportion is significantly higher than the 71% prevelance of a history of smoking among US men in the corresponding

**Table I** Prevalence of smokers among males

| | *Smokers* | *Odds ratio* | *P value* |
|---|---|---|---|
| Giant cell lung cancer cases | 93% (98/111) | | |
| *vs* | | | |
| US population 1965–80 | | | |
| Current smokers | 44% | 5.3 | <0.001 |
| Ever smokers | 72% | 16 | <0.001 |

Correspondence: B. Ballard, Department of Pathology, University of Texas Medical Branch, Galveston, TX 77550, USA.
Received 3 March 1989; and in revised form 9 May 1989.

years 1965–1980. Such an elevated proporiton of smokers among these giant cell carcinoma cases is good evidence that this form of lung cancer is causally related to smoking, as are all other histological types that have been adequately studied thus far (US Dept of Health & Human Services, 1982; International Agency for Research on Cancer, 1986). Comparison of this significantly higher proportion of smoking among these cases also implies that the relative risk for giant cell carcinoma of the lung is at least 5.3. This estimate is comparable to that for other histological types of lung cancer.

### References

BENSEL, W.L. & ISAHK, K.G. (1961). Giant cell carcinoma of the lung. *Am. J. Clin. Pathol.*, **35**, 435.

BRODERICK, P.A., CORVESE, N.L., LACHANCE, T. & ALLARD, J. (1975). Giant cell carcinoma of lung: a cytologic examination., *Acta Cytol.*, **19**, 225.

DAILEY, J.E. & MARCUSE, P.M. (1969). Gonadotropin secreting giant cell carcinoma of the lung. *Cancer*, **24**, 388.

DEANGELIS, C.E., ZARROW, E. & SOCHAN, O. (1961). Giant cell carcinoma of the lung: report of a case. *Am. Pract. Digest. Treat.*, **12**, 663.

FLANAGAN, P. & ROECKEL, I.E. (1964). Giant cell carcinoma of the lung: anatomic and clinical correlation. *Am. J. Med.*, **36**, 214.

FRIEDBERG, E.C. (1965). Giant cell carcinoma of the lung: a dedifferentiated adenocarcinoma. *Cancer*, **18**, 259.

GAJARAJ, A., JOHNSON, T.H. & FEIST, J.H. (1971). Roentgen features of giant cell carcinoma of the lung. *Am. J. Roentgenol. Radiat. Ther.*, **111**, 489.

GUILLAN, R.A. & ZELMAN, S. (1966). Giant-cell carcinoma of the lungs: an analysis of 12 cases. *Am. J. Clin. Pathol.*, **46**, 427.

HATHAWAY, B.M., COPELAND, K. & GURLEY, J. (1969). Giant cell adenocarcinoma of the lung: report of 21 and analysis of 139 cases. *Arch. Surg.*, **98**, 24.

HELLSTROM, H.R. & FISHER, E.R. (1963). Giant cell carcinoma of lung. *Cancer*, **16**, 1080.

HORIE, A. & OHTA, M. (1981). Ultrastructural features of large cell carcinoma of the lung with reference to the prognosis of patients. *Human Pathol.*, **12**, 423.

INTERNATIONAL AGENCY FOR RESEARCH ON CANCER (1986). *IARC monograph on the evaluation of the carcinogenic risk of chemicals to humans. Volume 38: Tobacco smoking*, p. 221. IARC: Lyon.

KALLENBERG, F. & JAQUE, J. (1979). Giant-cell carcinoma of the lung: clinical and pathological assessment. *Scand. J. Thorac. Surg.*, **13**, 343.

KENNEDY, A. (1969). Pathology and survival in operable cases of giant-cell carcinoma of the lung. *J. Clin. Pathol.*, **22**, 354.

LERNER, H.J. (1967). Giant cell carcinoma of the lung: review of literature and report of five cases. *Arch. Surg.*, **94**, 891.

MATSUO, K., IRIE, J., TSUCHIGAMA, H., NAKANO, M. & NAKATA, T. (1986). A high-graded malignancy bronchial mucoepidermoid carcinoma with features of giant cell carcinoma. *Acta Pathol. Jpn.*, **36**, 293.

NAIB, Z.M. (1961). Giant cell carcinoma of the lung: cytological study of the exfoliated cells in sputa and bronchial washings. *Dis. Chest*, **40**, 69.

NASH, A.D. & STOUT, A.P. (1958). Giant cell carcinoma of the lung: report of 5 cases. *Cancer*, **11**, 369.

PFEFFER, J.M. & STOVIN, P.G.I. (1978). Tumour production of alkaline phosphatase in a patient with giant-cell carcinoma of bronchus. *Thorax*, **33**, 261.

PFITZER, P. & KNOBLICH, P.G. (1975). Giant carcinoma cells of bronchiogenic origin. *Acta Cytol.*, **12**, 256.

RAZZUK, M.A., LYNN, J.A., KINGSLEY, W.B., RACE, G.J., URSCHEL, H.C. & PAULSON, M.D. (1970). Giant cell carcinoma of the lung. *J. Thorac. Cardiovas. Surg*, **59**, 574.

ROTHMAN, K.J. & BOICE, J.D. (1979). *Epidemiologic Analysis with a Programmable Calculator*, p. 31. US Govt Printing Office: Washington.

SHIN, M.S., JACKSON, L.K., SHELTON, R.W. & GREENE, R.E. (1986). Giant cell carcinoma of the lung: clinical and roentgenographic manufestations. *Chest*, **89**, 366.

THOMAS, C. (1962). Das Riesenzellcarcinom der Lunge. *Frankfurt. Zeit. Pathol.*, **72**, 302.

US DEPARTMENT OF HEALTH AND HUMAN SERVICES (1983). *The Health Consequences of Smoking: Cardiovascular Disease*, p. 363. US Govt Printing Office: Washington.

US DEPARTMENT OF HEALTH AND HUMAN SERVICES (1982). *The Health Consequences of Smoking: Cancer.* US Govt Printing Office: Washington.

WANG, N.S., SEEMAYER, T.A., AHMED, M.N. & KNOACK, J. (1976). Giant cell carcinoma of the lung. *Human Pathol.*, **7**, 3.

YESNER, R. & CARTER, D. (1982). Pathology of carcinoma of the lung: changing patterns. *Clin. Chest. Med.*, **3**, 257.